# Pushing forward: exploring the impact of the sitting position on muscle activation patterns and force generation during paralympic sit-cross-country skiing

**DOI:** 10.3389/fspor.2024.1441586

**Published:** 2024-09-11

**Authors:** Leonie Hirsch, Hatim Barioudi, Dominic Wintergerst, Ralf Rombach, Walter Rapp, Thomas Felderhoff, Natalie Mrachacz-Kersting

**Affiliations:** ^1^ Department of Neuroscience, Albert-Ludwigs Universität, Institute of Sports and Sports Science, Freiburg, Germany; ^2^ Faculty of Information Technology, Dortmund University of Applied Sciences and Arts Dortmund, Dortmund, Germany; ^3^ Deutscher Behindertensportverband und Nationales Paralympisches Komitee (DBS) e.V., Frechen, Germany; ^4^ Olympic Training Center Freiburg-Hochschwarzwald, Freiburg, Germany; ^5^ BrainLinks-BrainTools Center, IMBIT, Albert-Ludwigs University of Freiburg, Freiburg, Germany

**Keywords:** paralympic cross-country sit-skiing, sitting position, trunk control, muscle activation pattern, electromyography

## Abstract

Paralympic cross-country sit-skiing is a discipline of the Paralympic Winter Games where athletes use a specialized sledge. Athletes are classified into different groups according to their functional abilities. The double poling technique is used to push the sledge forward and generate speed. Different sitting positions in the sledge are used based on the individual impairment. To date there is no data available on the effects of these different positions on muscle activation patterns. The aim of this study was to analyze the muscle activation patterns of the trunk and upper body muscles in relation to the poling force. Nine Able-bodied athletes were tested on a treadmill at submaximal speed in three sitting positions for 4 min in a flat and uphill condition. Sitting positions included a “knee-high” position, a “knee-low” position, and a “neutral” position with the sitting platform parallel to the ground. Unilateral pole forces and surface EMG from three trunk muscles, two upper limb muscles, and one lower limb muscle were recorded simultaneously on the dominate side. Data were segmented into individual cycles and mean values and standard deviations calculated for each subject and condition. Statistical analyses, including a Friedman test and Bonferroni correction, were applied to examine significant differences across different sitting positions*.* Our findings demonstrate that while certain muscle groups such as the erector spinae and triceps show consistent patterns of activation across different sitting positions, there is considerable variability among individual athletes, suggesting individualized strategies for task execution. Overall, force application was most efficient in the “knee low” position with 691.33 ± 148.83 N and least efficient in the “knee high” position with 582.81 ± 115.11 N. Testing impaired athletes will be the next step in understanding the neurophysiological aspects of the poling movement. This experimental protocol provides a basis for understanding the movement of paralympic cross-country sit-skiing in greater depth.

## 1 Introduction

Over the last 20 years, paralympic sports have become increasingly popular and can be seen as a source of inspiration and a way to take part in elite sport. This surge in popularity has led to more people with physical limitations participating in paralympic sport. Sport-specific equipment, such as sledges for skiing, have helped facilitate their involvement in inclusive activities (1, 2).

Cross-country Sit-skiing (XCSS), characterized as an aerobic endurance sport (3, 4), necessitates effective movement patterns using the poling technique for racing on diverse snow conditions, turning on curves, and maintaining a high energy level during a race (5).

XCSS was introduced during the IV Winter Paralympic Games in 1988, offering a platform for athletes with a wide range of disabilities. Amputations, cerebral palsy, spinal cord injuries, visual impairments, and various other orthopaedic and neurological impairments are included (1). To promote fair competition, athletes with diverse impairments are classified into five classes [Locomotor Winter (LW) 10, LW10.5, LW11, LW11.5, and LW12] based on their functional capabilities (2–8). LW12-classified athletes can fully control their trunk, allowing a sitting position with greater range of motion achieved by lowering the knees below the level of the hips. Athletes classified as LW10 have the most severe impairment, and lack of trunk control. To move efficiently, these athletes adopt a sitting position where the knees are higher than the hips to prevent the upper body from falling forward.

Although there is a growing interest in paralympic winter sports there is a noticeable lack of scientific research on the neurophysiological aspects of XCSS (9). This research gap is all the more noteworthy given the burgeoning population of athletes actively engaging in paralympic sports (2). In most cases the sledge is individually made, and any mistake in construction can result in high costs. By designing an adjustable sledge, novices can test different sledge configurations and therefore the risk of building a not well adapted sledge for the individuals needs is reduced (9). Understanding whether an athlete can adopt a position that generates larger propulsive force is advantageous (8, 10). This might also lead to different classification due to the fact that muscle activation patterns during poling in the sledge are not included into the classification schema yet. Furthermore, the experience of the German national coach, who also contributed to this study, showed that, through training alone, some athletes who initially required a high knee position have improved trunk control and may later use a different, more advanced position.

The XCSS technique involves athletes sitting on a sledge and pushing themselves forward by impulsive impacts of the skiing poles. This mirrors the double poling technique used by able-bodied skiers (1, 7, 11). The athletes’ individual impairments greatly affect the sitting position as well as the execution of the movement. Different siting positions may therefore have different requirements and show divergent muscular activation patterns. This understanding is essential to guarantee fair competition and the development of evidence-based classification systems (7, 8). The purpose of this study was to characterize the intermuscular activation patterns during the forward propulsion of XCSS and to compare these patterns across different seating positions. We hypothesize that the KL position will result in improved performance due to a more effective use of the trunk muscles than the NT and KH positions. By thoroughly investigating these patterns, we aim to contribute valuable knowledge that can enhance individual athlete performance, improve training protocols, and support the development of an evidence-based classification system in this growing and exciting sport.

## 2 Materials and methods

### 2.1 Participants

Nine able-bodied athletes (24.5 ± 3.93 years; 179.12 ± 3.76 cm; 69.25 ± 7.36 kg, 6 male and 3 female) experienced in the double poling technique participated in the experiment. The sample size was determined based on previous literature (3, 8, 12). Participants were pre-informed about the protocol and the measurement tools with the possibility to withdraw from the measurements at any time and signed an informed consent. The study was approved by the Ethics Committee of the Albert-Ludwigs-University of Freiburg (Application no. EK-Freiburg: 23-1343-S2).

### 2.2 Experimental design and protocol

**Figure 1:**
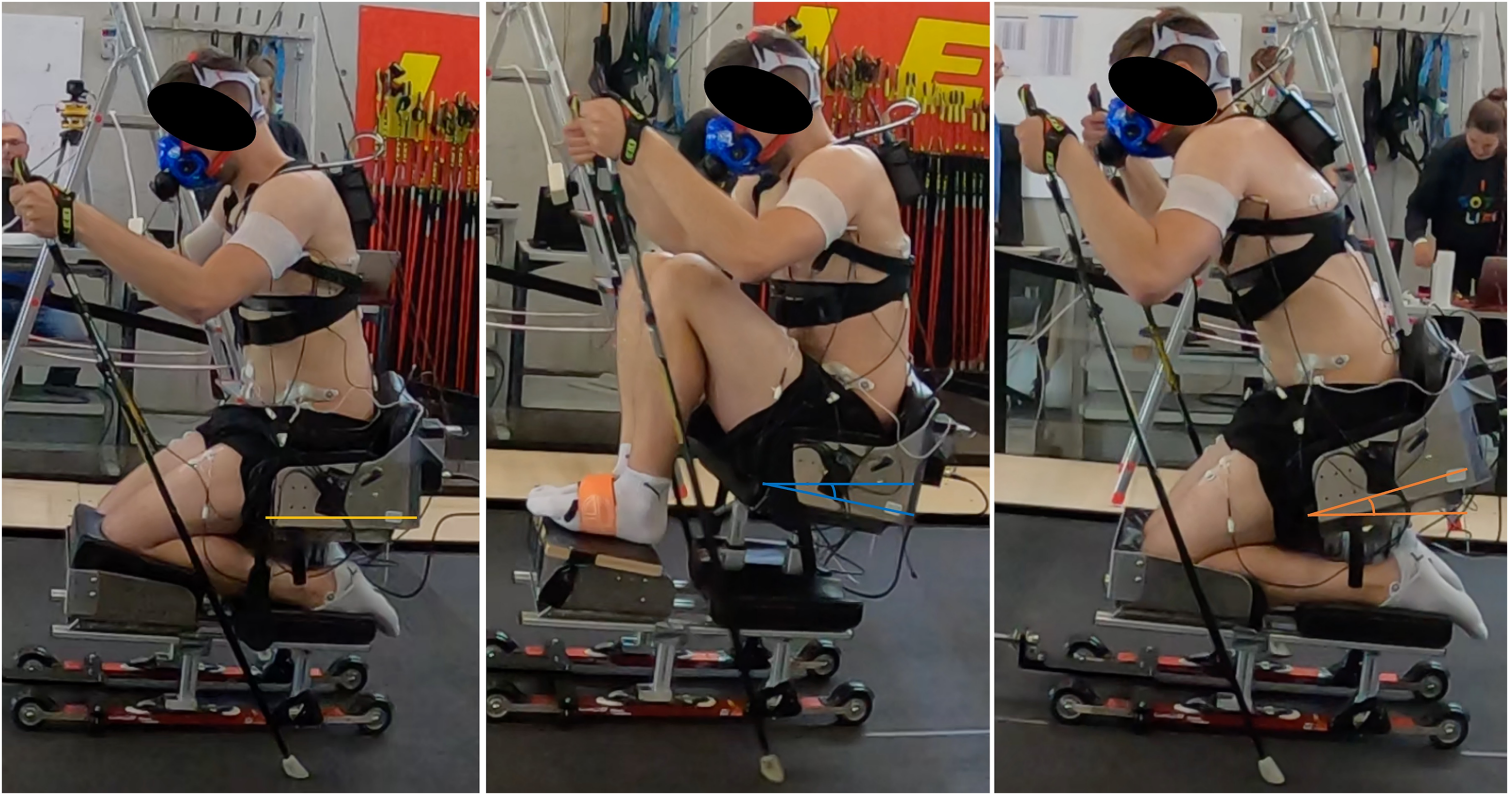
The three sitting positions: neutral (left), knee high (middle) and knee low (right).

**Figure 2:**
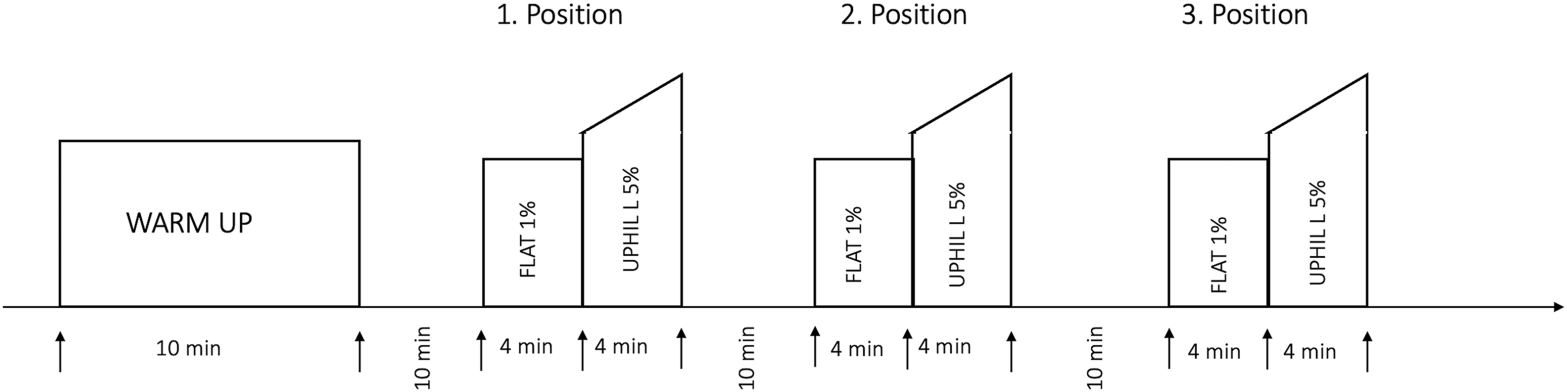
Performed protocol by the able-bodied athletes in three sitting positions: NT, KH, KL at submaximal individually chosen speed.

All tests were performed on a treadmill (Motek medical, Amsterdam, Netherlands) with a wheel-based adjustable sledge. Three positions of the sitting position were defined as knee-high (KH), neutral (NT) and knee-low (KL), based on the different classes defined in the Para Nordic guidelines for sit skiing (5, 13). In order to standardize the three sitting positions, the tilt of the sitting platform was fitted with a goniometer at an angle of −10° (KH), 0° (NT), + 15° (KL) for each condition (Figure 1). Participants were tested in all sitting positions using the double poling technique in a randomized order. The protocol consisted of a flat condition and an uphill condition. The flat condition was performed at 1% incline simulating air resistance and therefore the higher energy costs of outdoor performance (14). Each sitting position was tested for four minutes with no incline (flat) directly followed by 4 min with a 5% incline (uphill). Each athlete was instructed to select an individual speed that they could easily maintain for the flat and uphill condition separately. To make sure that all participants perform on an even level of Intensity they were instructed to choose the velocity according to the value 12 on the Borg Rating of Perceived Exertion (RPE) scale (15, 16). The velocity was tested during the Warm-up using the NT sitting position. In the following, all positions were tested with a ten-minute break in between each condition. The protocol is visualized in Figure 2. This paper will focus on the different activation patterns between the three positions in the flat condition.

Before testing, all participants were weighed, measured, and electrodes for the EMG measures were applied. The knee pad and sitting platform of the sled were adjusted to the anthropometric features of the participants and the sitting position.

### 2.3 Measurements of force and muscle activity

**Figure 3:**
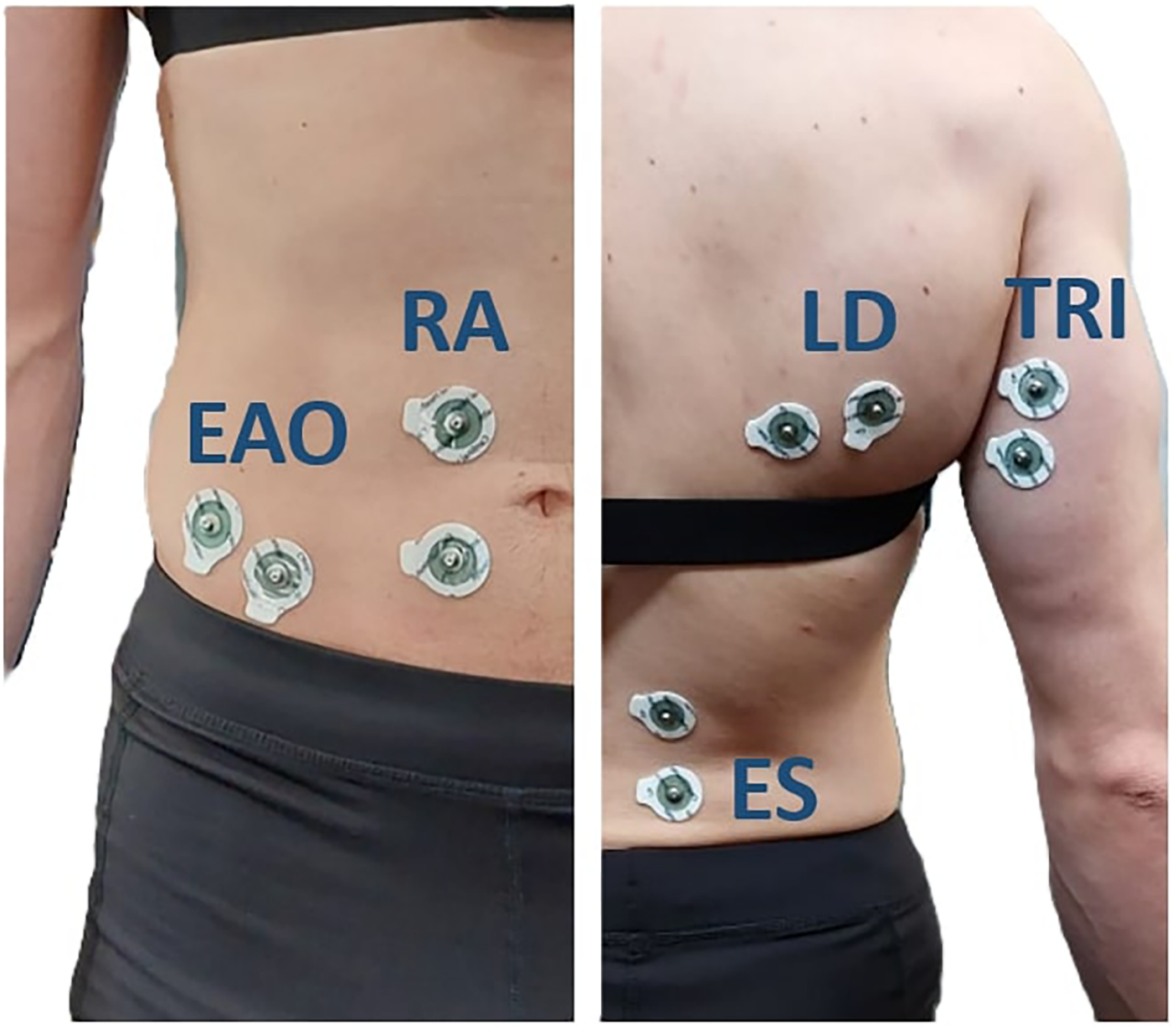
Positioning of surface EMG electrodes: M. erector spinae (ES), M. rectus abdominis (RA), M. external abdominal obliques (EAO), M. triceps brachii (TRI), M. latissimus dorsi (LD).

Pole forces and unilateral surface electromyography (EMG) were recorded simultaneously from three trunk muscles, two upper limb muscles and one lower limb control muscle. Bipolar surface electromyography was recorded using the Sessantaquattro wireless system from OT Bioelettronica s.r.l. (Torino, Italy). Sampling frequency of the surface electromyography data was 2,000 Hz. The Data was transmitted to a laptop and analysed afterwards. Before the placement of the electrodes skin was shaved if necessary and cleaned with alcohol. Electrodes were placed in the direction of the muscle fibre on the muscle belly according to literature (17). Since the double poling technique is performed symmetrically, surface electromyography was measured only on the dominant side (18). Muscles involved in the double poling movement were selected based on previous literature (5, 19, 20) and included: M. erector spinae (ES), M. rectus abdominis (RA), M. external abdominal obliques. (EAO), M. triceps brachii (TRI), M. latissimus dorsi (LD) and M. rectus femoris (RF). The Rectus femoris was measured as a “control muscle” to ensure that the participants performed the DP movement without using the lower limbs and therefore will not be analyzed further. The reference Electrode was placed on the anterior superior iliac spine. The placement of the electrodes is shown in Figure 3.

At the bottom of the ski pole on the dominant side, a force sensor (Type: KD40S, Manufacturer: HKM-Messtechnik GmbH, Freiburg, Germany) was installed to detect ground contact and record the exerted force. The force signal was connected via BNC connectors to the Sessantaquattro system by OT Bioelettronica s.r.l. (Turin, Italy) and recorded synchronously with the EMG measurement at a sampling rate of 2,000 Hz.

### 2.4 Data processing and statistical analyses

The EMG data were filtered with a 4th order digital Butterworth filter with cut-off frequencies from 10 Hz to 350 Hz. Additionally, a second-order recursive digital notch filter was applied to suppress the 50 Hz and all harmonics. A Root Mean Square envelope with a smoothing window of 100 ms was then calculated to complement the EMG signal filtering. In addition, a fourth-order low-pass filter with a cut-off frequency of 30 Hz was applied to the force data to obtain a clear and uniform pattern while minimizing interfering noise. The filtered data were then segmented into individual cycles using a threshold method based on the signal from the force sensors attached to the ski pole. The start of the poling phase was defined as 0% of a cycle, which corresponds to the moment the ski pole touches the ground, and the end of the recovery phase was defined as 100% of the cycle, which corresponds to the next Impact of the pole on the ground. Since the total time and thus data points differed for consecutive cycles within and also across participants, the filtered data for each cycle were resampled to 2,000 sampling points per cycle. Additionally, the amplitudes of the EMG signals for each muscle and each individual participant, were normalized to the maximum quantified in the test trials.

In a first step, a descriptive analysis was performed by calculating and analyzing the mean and standard deviation of all cycles for each muscle, subject and condition. On average these were based on a minimum of 60 individual cycles. Furthermore, the EMG onsets and offsets within one cycle of all muscles were calculated using an adaptive threshold approach (21). In addition, for a quantitative description, the following characteristics were extracted for each subject and condition: the mean value of the maximum force applied, the cycle duration, the frequency of poling and the percentage of time spent in the pole and recovery phase.

Due to the limited dataset, the Friedman test, a non-parametric statistical test, was chosen. The hypothesis under investigation was whether there are significant differences in the EMG activity of individual muscles during the three sitting positions. For this purpose, four features were examined: the maximum amplitude during the poling phase and the recovery phase, as well as the overall EMG activity during poling and recovery phases. The total EMG activity was calculated as the area under the EMG curve. Additionally, a Bonferroni correction was applied, setting the critical level at 0.0125 instead of 0.05 (*p* < 0.0125).

## 3 Results

### 3.1 Cycle characteristics

Table 1 presents the cycle characteristics and standard deviation for all sitting positions and participants. The parameters are presented descriptively as single-person data means and standard deviations. The poling cycle is divided into two phases: the poling phase (PP), where force is applied to the ground, and the recovery phase (RP), where the poles are brought back to the front. Among the participants, the cycle duration was longer in the KL position (1.83 ± 0.31 s) and attained the lowest values in the KH position (1.64 ± 0.61 s). Furthermore, the poling frequency decreased with a higher cycle duration in KL from 39.32 ± 7.17 to 33.68 ± 5.78 cycles per minute. The cycle length showed the highest values in the KL position (1.76 ± 0.33 m) and the lowest in the KH position (1.51 ± 0.27 m).

**Table 1:** Comparison of mean cycle characteristics and standard deviation for all sitting positions and participants in the KL (orange), NT (yellow) and KH (blue) position.

** **	P05	P06	P09	P10	P11	P12	P13	P14	P16
Force (*N*)	488 ± 89	491 ± 103	739 ± 151	544 ± 113	602 ± 156	567 ± 92	427 ± 6	775 ± 156	612 ± 113
Cycle duration (s)	1.53 ± 0.12	1.15 ± 0.20	2.30 ± 0.24	1.78 ± 0.23	1.35 ± 0.37	1.453 ± 0.49	1.72 ± 0.22	1.51 ± 0.21	1.42 ± 0.12
Poling phase (%)	40.51	36.76	48.43	45.52	35.58	36.65	55.37	30.76	33.48
Recovery phase (%)	59.49	63.24	51.57	54.48	64.42	63.35	44.63	69.24	66.52
Cycle length (m)	1.84	1.38	1.80	1.30	1.89	1.60	1.38	1.21	1.28
Poling frequency (cycles/min)	39.11	52.04	26.71	33.71	44.35	41.26	34.82	39.63	42.25
Force (*N*)	572 ± 102	527 ± 98.30	883 ± 168	915 ± 175	671 ± 124	678 ± 111	512 ± 108	813 ± 164	651 ± 125
Cycle duration (s)	1.88 ± 0.23	1.31 ± 0.12	2.29 ± 0.35	1.81 ± 0.26	1.64 ± 0.86	1.60 ± 0.24	2.02 ± 0.22	2.05 ± 0.28	1.83 ± 0.13
Poling phase (%)	24.73	35.96	38.66	40.32	28.53	30.66	47.50	42.5	31.95
Recovery phase (%)	75.27	64.04	61.34	59.68	71.47	69.34	52.497	57.5	68.05
Cycle length (m)	2.25	1.57	1.83	1.27	2.29	1.76	1.61	1.64	1.65
Poling frequency (cycles/min)	31.97	45.91	26.19	33.15	36.65	37.43	29.75	29.33	32.79
Force (*N*)	523 ± 47	467 ± 72	773 ± 130	813 ± 173	647 ± 98.4	634 ± 99.67	496 ± 88.22	723 ± 163	603 ± 89
Cycle duration (s)	1.74 ± 0.23	1.11 ± 0.18	2.02 ± 0.24	1.76 ± 0.13	1.46 ± 0.24	1.59 ± 0.22	1.92 ± 0.38	1.52 ± 0.21	1.73 ± 0.18
Poling phase (%)	28.93	38.36	44.61	44.81	42.32	34.82	49.23	41.56	32.21
Recovery phase (%)	71.07	61.64	55.39	55.19	57.68	65.18	50.77	58.44	67.79
Cycle length (m)	2.09	1.34	1.62	1,23	2,35	1,75	1.54	1.21	1.56
Poling frequency (cycles/min)	34.48	53.91	29.67	34.09	35.71	37.66	31.25	39.53	34.68

The average maximum force across all participants was highest in the KL (691.33 ± 148.83 N) position and lowest in the KH position (582.81 ± 115.11 N), while for the NT position values were at 644.5 ± 123.32 N. The maximum peak occured at two thirds after the initial ground contact. In KL, the duration of the poling phase had the lowest percentage of the whole cycle (35.71 ± 7.31%), whereas in KH it had the longest (40.4 ± 7.95%). This relationship was reversed in the recovery phase, with KL attaining the highest percentage of 64.35 ± 7.31% and KH the lowest with 59.66 ± 7.95%.

### 3.2 Poling force

**Figure 4:**
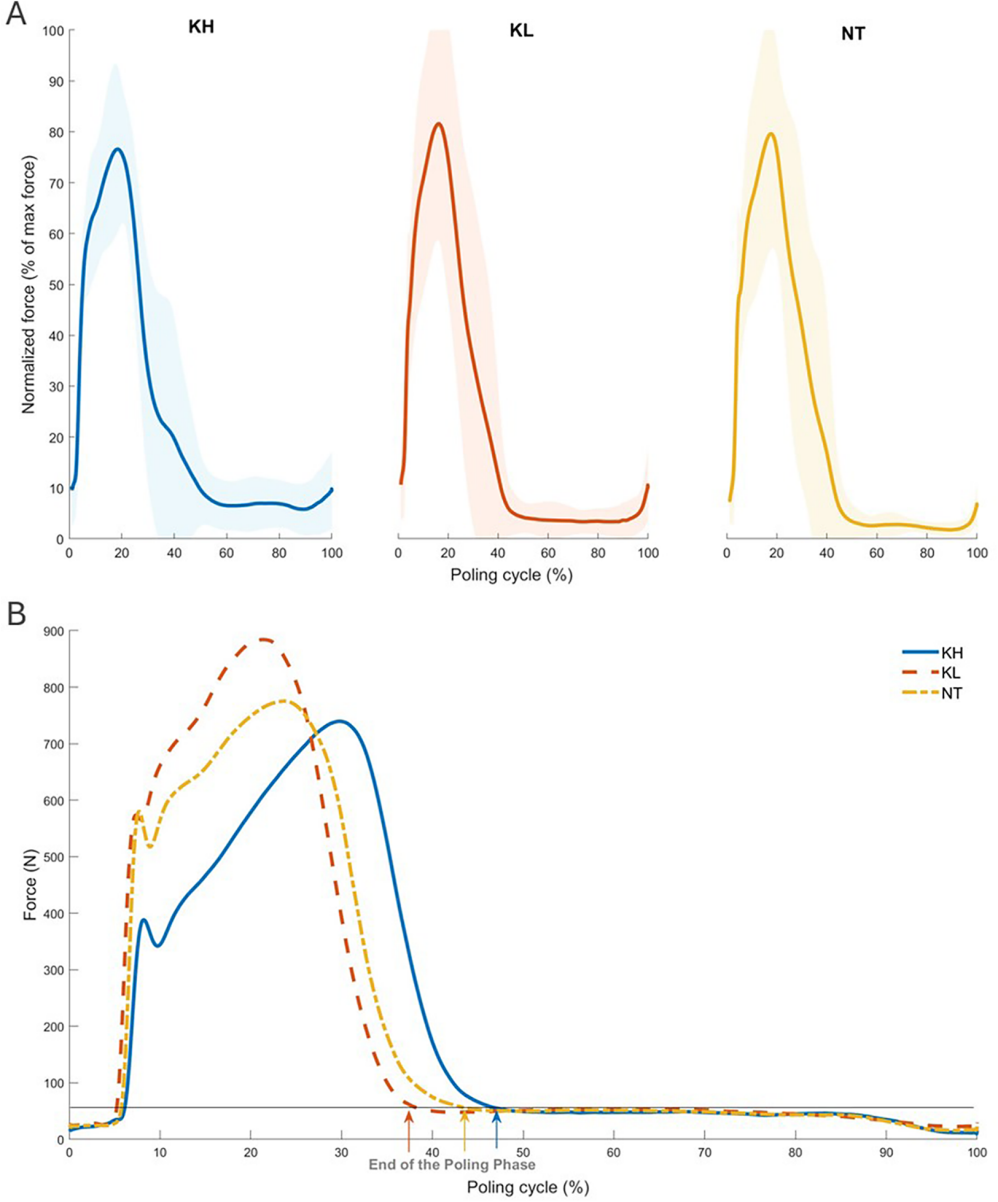
**(A)** Mean force production profiles of all participants in KL (orange), NT (yellow) and KH (blue) position and the standard deviation area is presented. **(B)** Mean force production profiles of all participants in KL (orange), NT (yellow) and KH (blue). The arrows show the end of the poling phase in the associated color.

Figure 4 shows the average force production profiles in all three sitting positions. Force profiles were similar between the sitting positions and participants. The force generation profiles varied between the positions, with differences observed in the poling phase. In all positions, the initial contact generated a initial increase in force, leading to a first steep slope with an intermediate less steep gradient after the initial contact. The force value then increased towards the maximum force value, occurring first in KL, then in NT, and lastly in KH. The average gradient during the increasing phase was steepest in KL, with a value of 3.53. This is higher compared to NT, which had an average gradient of 2.99, and KH, which had an average gradient of 1.80. After reaching the maximum force value, the force decreased towards the end of the poling phase. The KH position had the longest force application time (poling phase) with a value of 48.43% of the cycle, while KL had the shortest with 38.66% of the cycle. As a result, the recovery time was longest in KL (61.34%) and shortest in KH (51.57%). The maximum pole force was highest in the KL position (883 N), and lowest in the KH position (739 N).

### 3.3 Qualitative description of individual muscle activation patterns

**Figure 5:**
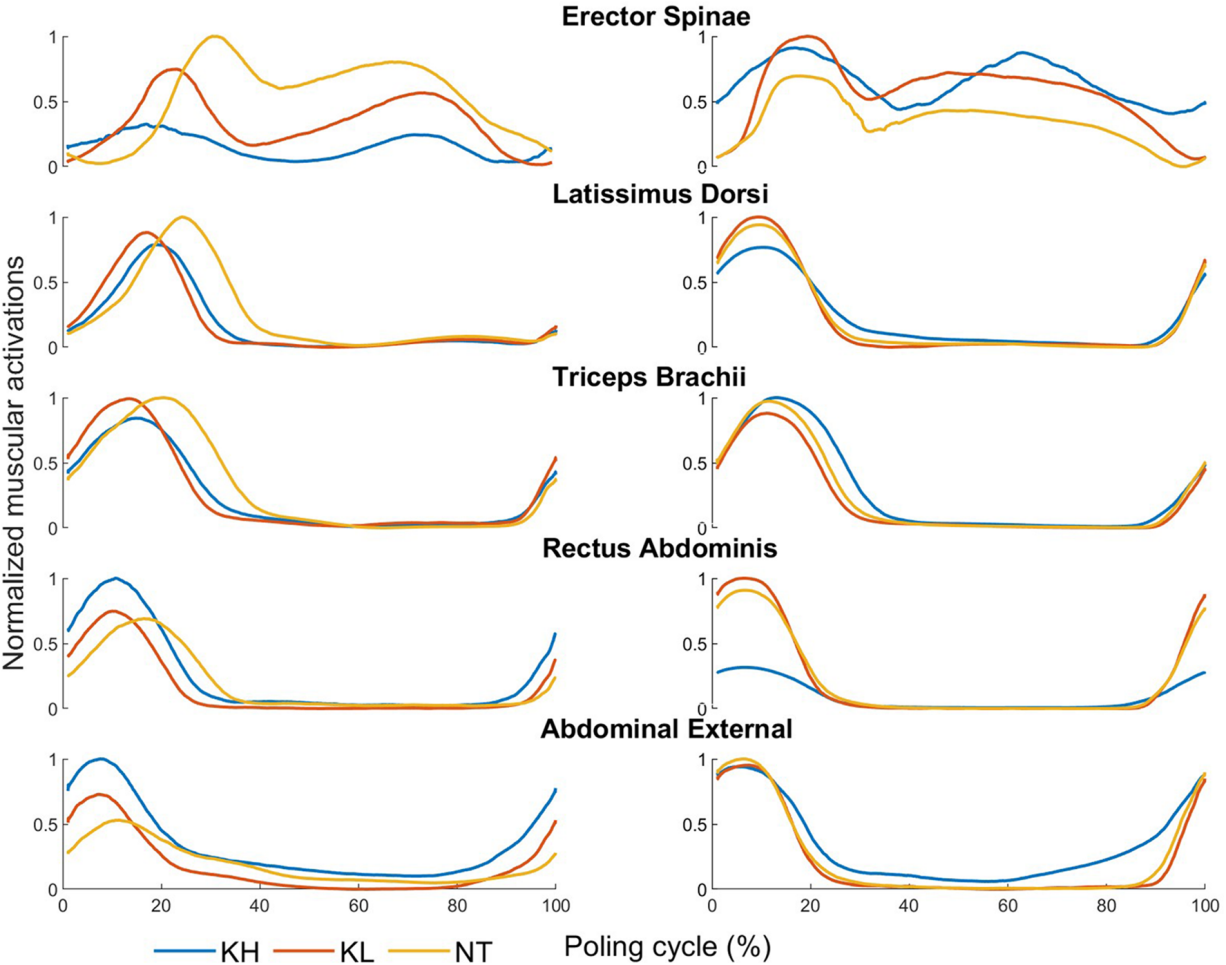
Comparison of mean surface electromyography profiles of all tested muscles and poling (grey) and recovery phase (white) of subjects P05 (left) and P03(right) in the KL (orange), NT (yellow) and KH (blue) position.

Figure 5 displays the muscle activation patterns of two participants, illustrating the complex interplay of muscle dynamics during different phases of the poling cycle. The mean values of muscle onsets and offsets for each muscle are presented in Table 2 for all sitting positions. Muscle onsets and offsets of single participants are presented in Supplementary Table 4 in the supplementary material. Notably, the erector spinae show a distinct double-peak activation pattern in all positions: a first peak during the poling phase and a second during the late recovery phase. The erector spinae activation in the NT position begins at 4.44% position during the poling phase and continues until 90.70% in the recovery phase, underscoring its crucial role throughout the poling cycle. This activation pattern looks similar to the KL position. Interestingly, the erector spinae is active during the entire recovery phase. This is not observed in other muscles. In the KH position, there is a period of inactivity for the erector spinae between 35.19% and 47.9% of the cycle, highlighting position-specific muscle activation patterns. Activation details for the other included muscles show that they are mainly active during the poling phase. The activation of the other muscles starts at the end of the poling phase in all positions. This pre-activation emphasizes their role in force generation stabilization of the upper body. For latissimus dorsi and triceps brachii the onset timing is very similar between all positions. For the rectus abdomis and the external abdominal obliques, the activation occurs a bit earlier in the recovery phase.

**Table 2:** Comparison of the mean and standard deviation of muscle on-sets and off-sets of the ES, erector spinae; LD, latissimus dorsi; TRI, triceps brachii; RA, rectus abdominis; AEO, abdominal external obliques.

	ES				LD		TRI		RA		AEO	
	Onset 1	Offset 1	Onset 2	Offset 2	Onset	Offset	Onset	Offset	Onset	Offset	Onset	Offset
KH	14.34 ± 31.6	35.19 ± 5.1	4.,9 ± 5.7	79.94 ± 8.6	97.76 ± 1.3	31.20 ± 4.9	96.38 ± 1.3	33.32 ± 3.8	93.50 ± 3	27.18 ± 5	88.90 ± 4.69	30.52 ± 5.61
KL	5.08 ± 2.1	90.05 ± 4.3	-	-	97.84 ± 1.1	26.38 ± 4.4	96.82 ± 1.4	26.88 ± 4.0	95.53 ± 2.2	22.57 ± 5.6	91.80 ± 3.70	24.95 ± 6.26
NT	4.44 ± 2	90.70 ± 5.2	-	-	97.89 ± 1.2	30.25 ± 5.1	96.38 ± 1.8	31.24 ± 4.3	95.42 ± 2.5	26.98 ± 6.4	93.65 ± 3.33	30.14 ± 8.09

**Figure 6:**
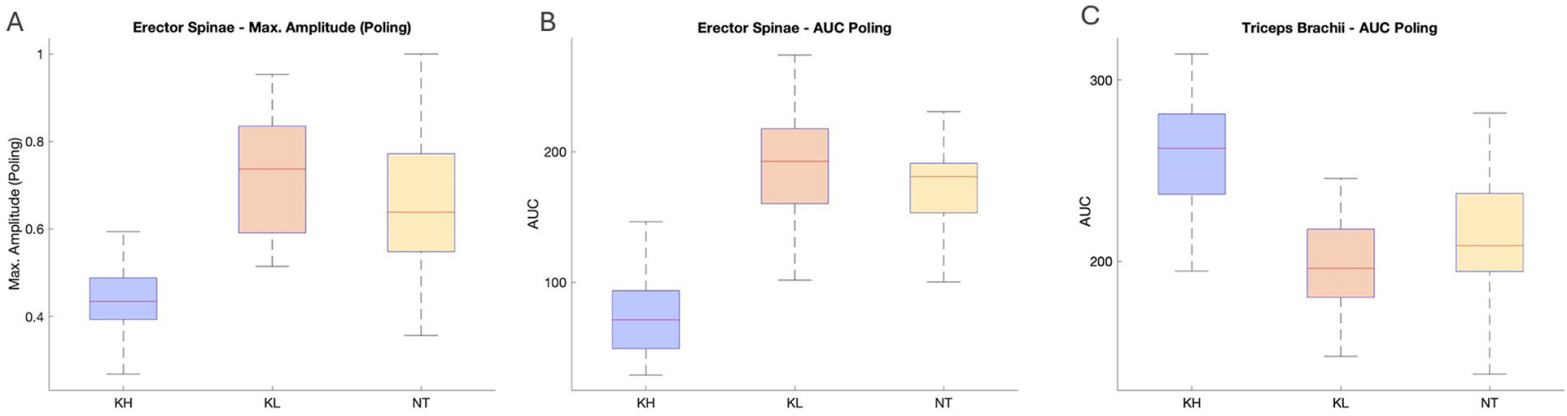
**(A)** Shows significant differences between the maximum amplitude in the PP in the erector spinae. **(B)** Shows significant differences in the area under the curve in the PP in the erector spinae (ES). **(C)** Shows significant differences in the area under the curve in the PP in the triceps brachii (TRI).

The Statistical analysis is presented in Table 3 and visualized in Figure 6. It reveals some significant differences in muscle activation across the three tested sitting positions. Notably, the erector spinae maximum EMG amplitude in the poling phase was significantly different (*p* = 0.0039, Figure 6A), indicating relevant differences in muscle activity depending on the sitting position. The area under the curve for the erector spinae was notably higher in the KL position compared to others (*p* = 0.00012, Figure 6B), highlighting how the position of the knee impacts muscle activity of the trunk muscles. The triceps brachii however, showed the highest area under the curve in the KH position (*p* = 0.00159, Figure 6C). This suggests different biomechanical demands between positions, with a more trunk involvement in KL and more work done by the arms in KH.

**Table 3:** Results of the Friedmann test between the KH (blue), KL (orange) and NT (yellow) position.

* *	ES	LD	TRI	RA	AEO
Max amp. (Poling)	<0.0125	0.137	0.15	0.441	0.404
Max amp. (Rec)	0.014	0.085	0.064	0.041	0.145
AUC poling	<0.0125	0.181	<0.0125	0.095	0.389
AUC recovery	0.061	0.181	0.016	0.014	0.921

The table in **(A)** presents results of the Friedman test between the individual muscles, for the maximum amplitude (Max. Amp.) and the Area under the curve (AUC). Significant values with the value *p* < 0.0125 are marked in red.

These findings underscore the complex and significant impact of sitting position on muscle activation patterns. This understanding is essential for optimizing performance and training in paralympic cross-country sit-skiing.

## 4 Discussion

This study aims to enhance our understanding of how muscle activation patterns vary with different sitting positions in sit-skiing. However, it also highlights the necessity for more comprehensive studies to elucidate these complex interactions. Differences between the sitting positions were visible. Overall, force parameters and muscle activation patterns differed between the KH and KL position, with significant variations for the erector spinae and triceps brachii.

### 4.1 Cycle characteristics

Previous studies have investigated characteristics of the poling cycle. These report that the KH position has the highest poling frequencies and shortest poling cycles, confirming our results (5, 22). Additionally, the poling phase has the highest percentage of the cycle length, resulting in a shorter recovery phase [(5), see Table 1]. The KH position exhibits the lowest values for both maximum generated force, cycle time and cycle length [(11, 22), see Table 1]. Our study thus supports existing research and the hypothesis that the Poling Cycle is less effective in the KH position due to lower force values and a shorter recovery phase between cycles. In contrast, in the KL position the highest force is applied and the recovery phase is the longest. These characteristics of the poling cycle show that the KL position is a better position to apply force effectively during double-poling in a sit ski (5).

### 4.2 Pole force

Pole force profiles exhibit a consistent pattern across the participants and sitting positions. The KL position yields the highest maximal force due to the extended range of motion, which allows for greater usage of trunk muscles (23). The active involvement of these muscles contributes to core stability, and the entire upper body is involved in the force transfer to the poles (23). The activated core muscles also enable the athlete to stabilize the core when applying force to the ground and work against the ground reaction forces (23). Additionally, the longer recovery phase in KL enables skiers to apply higher forces in subsequent cycles, resulting in sustained performance during a race (11). The lowest maximal force in KH is a result of the smaller range of motion. The position of the legs in front of the upper body constrains the momentum of the upper body, requiring greater force generation in the arms. Figure 6C) illustrates a more active engagement of the triceps brachii in KH which also present with a shorter recovery phase, which may lead to faster fatigue of the triceps brachii. When performing at the same submaximal speed, the force application time in KH is longer due to smaller force values.

### 4.3 Muscle activation patterns

Unlike the pole forces, muscle activation were participant specific with slight variation observed (see Table 2). Rosso et al. (24) describe the threefold role of the trunk in XCSS which includes trunk momentum, trunk position, and trunk stability. These factors influence the XCSS performance relevantly and will be used to interpret our findings. To understand the results regarding the muscle activation profiles it is important to note that the different durations of the poling phase can be assumed to result in different activation times of the muscles when comparing the three sitting positions.

The trunk momentum, vital for propulsive force generation, is created from flexion-extension movements transferring upper body momentum to the skiing poles (24). Analyzing the muscle activation patterns, flexion of the trunk in the first part of the poling cycle is visible with an activation of the rectus abdominis and the external abdominal obliques. The later following first peak of the ES activation could be explained as a counter movement to stop the forward momentum of the upper body. The second peak of the erector spinae could initiate the extension of the trunk in the recovery phase where the upper body is brought back into a vertical position. This movement profile was also described by Karczewska-Lindinger et al. (5) as a trunk flexion in the first two-thirds of the poling phase and which occurs in skiers in the LW 12 class and a less pronounced flexion in skiers in the LW 11 class (Less control of trunk muscles) (5). In the LW10 class (no control of the trunk) a trunk extension is visible during poling phase (5). The difference in the erector spinae activation between the sitting position is an inactive period of the erector spinae in the mid-cycle in KH, while being constantly active around 85% of the cycle in KL and NT. The part of the cycle where the erector spinae is “shut off” in the last part of the poling phase and the start of the recovery phase (poling phase ending at approximately 40% of the poling phase) may indicate that the thighs which are above hip level halt the upper body in the forward movement. They would in that case function as a stopping mechanism and the erector spinae is not needed to deaccelerate the upper body movement. This mechanism is especially used in athletes with no or very little trunk function in the LW10 and LW10.5 class which require upper body support (3, 25). Later in the cycle, the erector spinae is active again in the able-bodied athletes to maintain an upright positioning of the upper body as observed in the other two positions.

As described by Nielsen et al. (20), a proximo-distal muscle activation pattern is in standing elite cross-country skiers. The peak EMG value is first attained in the rectus abdominis and the external abdominal obliques and later in the poling phase by the latissimus dorsi and triceps brachii. The trunk muscles are active first, followed by the muscles int the upper body. This suggests that the XCSS poling movement is initiated by a flexion-extension pattern of the trunk, directly followed by a flexion-extension pattern in the elbow joint (12) which requires an eccentric-concentric moment of the triceps brachii. The activation of the triceps brachii and latissimus dorsi thus involves a stretch-shortening cycle (SSC) relevant for the propulsive force application in the DP movement (12, 18).

Trunk stability is imperative to maintain the upper body vertical in the sagittal body plane and to apply force homogenously on both sides. The recorded pre-activation may be a mechanism to ensure this. The pre-activation of the muscles at the end of the recovery phase could also be explained through the preparation of the neuromuscular system to create enhanced stiffness of the muscle-tendon system, to create higher muscular power for the initial pole-ground contact (12). The pre-activation has previously been shown to be a mechanism to store elastic energy in the muscle-tendon which can be used in dynamic eccentric movements such as exhibited in the triceps brachii and the latissimus dorsi (26). This supports the hypothesis that a SSC could be involved in the DP movement also in the seated position in the sledge.

The trunk position plays an important role in DP as the third parameter in the sledge (22), The main difference between the three tested positions is the Range of motion (ROM) of the upper body as a result of a larger spinal flexion in KH (3). Resulting in the effect that during the PP the athlete cannot lean the upper body to the front, creating an impulse momentum as in KL (22). Therefore, the propulsive force in KH is lower. To gain the same speed as the KL position athletes must apply force for a longer time and therefore have a shorter recovery phase. The significant results describing differences between the positions in the erector spinae could also be explained through the limited ROM in KH position: the upper body flexion in poling phase is limited and therefore the following extension is not as pronounced. In the KL and NT positions the knees are lower than the hip level. Therefore, a more pronounced trunk flexion is possible and used for force-impulsive generation. The erector spinae in that case as the antagonist straightens the upper body back up into the vertical position (5). Significant differences in the triceps brachii can be explained through a compensation mechanism for the limited trunk ROM. The triceps brachii has a higher level of activation to generate the same pole force to keep the same speed. Overall, the values of NT and KL are more similar to each other because the knees were below hip level in both positions.

### 4.4 Limitations

Although this study on muscle activation patterns in Paralympic cross-country skiing provided valuable insights, it is important to acknowledge its limitations. Due to the small sample size, our study is limited in its ability to generalize the findings across a broader population of able-bodied athletes and furthermore to impaired athletes. As already mentioned, the study included only able-bodied athletes, which limits a direct projection of our findings to impaired athletes. Experienced able-bodied athletes were specifically selected to ensure consistency and proficiency in the double poling technique across all participants. Furthermore, the recording of the rectus femoris muscle allowed us to monitor its activity, thus ensuring that movements were initiated from the upper body without lower leg actions. In addition, despite the participants’ familiarity with the double poling technique, sitting in a sled for the first time may have resulted in a learning curve during the experimental protocol. To mitigate this potential learning effect, we conducted a thorough warm-up phase and randomized sitting positions and participants. However, it is possible that participants felt more secure in the neutral sitting position, which may have restricted their use of the full range of motion in the KL sitting position. These limitations reduce the generalizability of the muscle activation patterns found due to differences in the ability to control the trunk between able-bodied and impaired athletes. Impaired athletes have a wide range of trunk abilities represented in the different classes of the classification system. Therefore, it will be important to test this protocol for different levels of impairment in the future.

## 5 Conclusion

This study investigated the differences in muscle activation patterns in Paralympic cross-country skiing, specifically in relation to sitting positions. The study builds upon performance differences in the double poling technique. Results reveal a consistent feature for differentiating between sitting positions and a high degree of interpersonal variability in muscle activation patterns among able-bodied participants. This highlights the individualized nature of movement, despite achieving similar force application outcomes in the end. Considering individual differences is crucial when determining the optimal sitting position for athletes, as highlighted by these diverse movement patterns. The limited range of motion in the KH position results in lower activation of the erector spinae and higher activation of the triceps brachii, which compensates for the restricted movement of the upper body as an impulse transmitter.

Our experimental protocol provides a foundational framework for future research involving Paralympic athletes. It provides a starting point for using electromyography data to assess trunk function and sled movement ability. Additionally, it has the potential to assess the optimal sitting position and provide athletes with personalized feedback on their poling efficiency in relation to their sitting position. Moving forward, it is important to extend this research to include impaired athletes and expand the testing to actual race conditions. Their unique biomechanical profiles may yield further insights into muscle activation patterns and sitting position optimization. By analyzing EMG data and refining our understanding of individual movement patterns, we can improve training and performance in this advancing the field of adaptive sports science.

## Data Availability

The raw data supporting the conclusions of this article will be made available by the authors, without undue reservation.

## References

[1] Gastaldi L Pastorelli S Frassinelli S. A biomechanical approach to paralympic cross-country sit-ski racing. Clin J Sport Med. (2012) 22(1):58–64. 10.1097/JSM.0B013E31824202D3 22222588

[2] Gastaldi L Mauro S Pastorelli S. Analysis of the pushing phase in paralympic cross-country sit-skiers—class LW10. J Adv Res. (2016) 7(6):971–8. 10.1016/j.jare.2016.10.003 27857844 PMC5099268

[3] Liu C Tian Y Zhou L Tian Z Sun G Yin J Upper limb isokinetic muscle strength predicts the performance in cross-country sit-skiing. Sci Rep. (2022) 12(1):6093. 10.1038/s41598-022-10103-4 35414091 PMC9002028

[4] Lund Ohlsson M Laaksonen M Laaksonen MS. Sitting position affects performance in cross-country sit-skiing. Eur J Appl Physiol. (2017) 117(6):1095–106. 10.1007/s00421-017-3596-y 28382550 PMC5427162

[5] Karczewska-Lindinger M Linnamo V Rosso V Gastaldi L Rapp W Vanlandewijck Y Force generation profiles of para-Nordic sit-skiers representing different physical impairments. J Sci Sport Exerc. (2021) 3(3):281–91. 10.1007/s42978-021-00117-1

[6] Pernot HFM Lannem AM Geers RPJ Ruijters EFG Bloemendal M Seelen HAM. Validity of the test-table-test for Nordic skiing for classification of paralympic sit-ski sports participants. Spinal Cord. (2011) 49(8):935–41. 10.1038/SC.2011.30 21537336

[7] Linnamo V Rapp W Lindinger SJ. Contribution of sport science to performance: Nordic skiing. In: Vanlandewijck YC, Thompson WR, editors. Training and Coaching the Paralympic Athlete. Oxford: John Wiley & Sons (2016). 10.1002/9781119045144.ch11

[8] Lajunen K Rapp W Ahtiainen JP Lindinger SJ Linnamo V. Effect of sitting posture on sit-skiing economy in non-disabled athletes. Front Sports Act Living. (2020) 2:44. 10.3389/fspor.2020.00044 33345036 PMC7739656

[9] Rombach R Rapp W. Analysis of support structures in paralympic Nordic skiing in international comparison-an exploratory study. In: Science and Skiing VI. (2014). 465–74.

[10] Rapp W Lindinger S Lappi T Ohtonen O Linnamo V. Force production, balance control and muscle activation in different sitting positions-pilot study for disabled sit sledge cross-country skiers. In: The 6th International Congress on Science and Skiing (ICSS); 2013 Dec 14-19; St. Christoph/Arlberg - Austria. Vol. 53. Maidenhead: Meyer & Meyer Verlag (2013).

[11] Bernardi M Janssen T Bortolan L Pellegrini B Fischer G Schena F. Kinematics of cross-country sit skiing during a paralympic race. J Electromyogr Kinesiol. (2013) 23(1):94–101. 10.1016/j.jelekin.2012.07.004 22902105

[12] Lindinger SJ Holmberg HC Müller E Rapp W. Changes in upper body muscle activity with increasing double poling velocities in elite cross-country skiing. Eur J Appl Physiol. (2009) 106(3):353–63. 10.1007/S00421-009-1018-5 19280214

[13] International Paralympic Committee. World Para Nordic skiing. World Para Nordic Skiing Classification Rules and Regulations. Bonn: International Paralympic Committee (2017). Available online at: www.WorldParaNordicSkiing.org

[14] Jones AM Doust JH. A 1% treadmill grade most accurately reflects the energetic cost of outdoor running. J Sports Sci. (1996) 14(4):321–7. 10.1080/02640419608727717 8887211

[15] Borg GA. Psychophysical bases of perceived exertion. Med Sci Sports Exercise. (1982) 14(5):377–81. 10.1249/00005768-198205000-00012 7154893

[16] Williams N. The Borg rating of perceived exertion (RPE) scale. Occup Med (Chic Ill). (2017) 67(5):404–5. 10.1093/occmed/kqx063

[17] Criswell E Cram JR, editors. Chapter 4. Cram’s Introduction to Surface Electromyography. 2nd ed. Sudbury, MA: Jones and Bartlett (2011). p. 341–51.

[18] Zoppirolli C Pellegrini B Modena R Savoldelli A Bortolan L Schena F. Changes in upper and lower body muscle involvement at increasing double poling velocities: an ecological study. Scand J Med Sci Sports. (2017) 27(11):1292–9. 10.1111/SMS.12783 27726202

[19] Holmberg HC Lindinger S Stöggl T Eitzlmair E Müller E. Biomechanical analysis of double poling in elite cross-country skiers. Med Sci Sports Exerc. (2005) 37(5):807–18. 10.1249/01.MSS.0000162615.47763.C8 15870635

[20] Nilsson J Tinmark F Halvorsen K Arndt A. Kinematic, kinetic and electromyographic adaptation to speed and resistance in double poling cross country skiing. Eur J Appl Physiol. (2013) 113(6):1385–94. 10.1007/S00421-012-2568-5 23229884

[21] Tabie M Kirchner EA. EMG onset detection—comparison of different methods for a movement prediction task based on EMG. Int Conf Bio Inspired Syst Signal Process. (2013):242–7. 10.5220/0004250102420247

[22] Rosso V Linnamo V Rapp W Lindinger S Karczewska-Lindinger M Vanlandewijck Y Simulated skiing as a measurement tool for performance in cross-country sit-skiing. Proc Inst Mech Eng Part P J Sports Eng Technol. (2019) 233(4):455–66. 10.1177/1754337119843415

[23] Rosso V Linnamo V Rapp W Lindinger S Karczewska-Lindinger M Vanlandewijck Y *Different sitting positions influence cross country sit skiers performance sitting position influence on force generation and cycle characteristics*. MEMEA 2018–2018 IEEE International Symposium on Medical Measurements and Applications, Proceedings. (2018). 10.1109/MEMEA.2018.8438670

[24] Rosso V Gastaldi L Rapp W Lindinger S Vanlandewijck Y Äyrämö S Balance perturbations as a measurement tool for trunk impairment in cross-country sit skiing. Adapt Phys Activ Q. (2018) 36(1):61–76. 10.1123/APAQ.2017-0161 30563347

[25] Tweedy SM Vanlandewijck YC. International paralympic committee position stand–background and scientific principles of classification in paralympic sport. Br J Sports Med. (2011) 45(4):259–69. 10.1136/bjsm.2009.065060 19850575

[26] Bosco C Tihanyi J Komi PV Fekete G Apor P. Store and recoil of elastic energy in slow and fast types of human skeletal muscles. Acta Physiol Scand. (1982) 116(4):343–9. 10.1111/J.1748-1716.1982.TB07152.X 7170997

