# Supplementary material for: Pushing forward: exploring the impact of the sitting position on muscle activation patterns and force generation during paralympic sit-cross-country skiing
Source: Front Sports Act Living. 2024 Sep 11;6:1441586. doi: 10.3389/fspor.2024.1441586 (PMC11457913; doi:10.3389/fspor.2024.1441586)
Supplement: Supplementary file 1 [file Table1.docx]

Supplementary Material

**Maximizing Potential: Exploring the Impact of the Sitting Position on Muscle Activation Patterns and Force Generation During Paralympic Sit-Cross-Country Skiing**

**Leonie Hirsch^1^, Hatim Barioudi^3^, Dominic Wintergerst^1^, Ralf Rombach^2^, Walter Rapp^2^, Thomas Felderhoff^3^ and Natalie Mrachacz-Kersting^1^**

*** Correspondence: Natalie Mrachacz-Kersting**: [natalie.mrachacz-kersting@sport.uni-freiburg.de](mailto:natalie.mrachacz-kersting@sport.uni-freiburg.de)

# Supplementary Table

**Table 4** Shows the means of muscle on-sets and off-sets of all participants of the erector spinae (ES), latissimus dorsi (LD), triceps brachii (TRI), rectus abdominis (RA), abdominal external obliques (AEO).

| KH | ES | | | | LD | | TRI | | RA | | AEO | |
| --- | --- | --- | --- | --- | --- | --- | --- | --- | --- | --- | --- | --- |
|  | **Onset 1** | **Offset 1** | **Onset 2** | **Offset 2** | **Onset** | **Offset** | **Onset** | **Offset** | **Onset** | **Offset** | **Onset** | **Offset** |
| P01 | 1.04 | 31.75 | 45.27 | 71.42 | 97.94 | 27.89 | 97.25 | 34.42 | 93.95 | 24.62 | 91.84 | 26.24 |
| P02 | 1.68 | 38.53 | 47.70 | 74.69 | 95.04 | 32.69 | 94.55 | 33.58 | 88.06 | 26.65 | 87.95 | 28.14 |
| P03 | 98.48 | 37.74 | 58.44 | 84.94 | 98.91 | 30.61 | 96.97 | 31.25 | 96.68 | 26.80 | 83.90 | 33.04 |
| P04 | 7.88 | 39.82 | 47.10 | 93.66 | 99.23 | 38.19 | 95.93 | 37.84 | 95.44 | 36.75 | 93.46 | 34.77 |
| P05 | 3.82 | 32.29 | 46.17 | 81.97 | 97.22 | 23.48 | 97.82 | 27.00 | 92.07 | 20.06 | 83.06 | 19.22 |
| P06 | 1.99 | 32.05 | 41.51 | 78.11 | 98.86 | 30.46 | 94.75 | 29.52 | 90.93 | 27.59 | 83.50 | 36.90 |
| P07 | 5.30 | 44.18 | 56.07 | 77.16 | 98.36 | 31.51 | 98.16 | 31.81 | 97.37 | 33.13 | 88.14 | 34.58 |
| P08 | 5.50 | 28.83 | 42.39 | 90.22 | 97.96 | 38.44 | 96.78 | 38.19 | 94.60 | 23.92 | 93.01 | 33.93 |
| P09 | 3.37 | 31.50 | 47.30 | 67.26 | 96.32 | 27.52 | 95.23 | 36.26 | 92.42 | 25.06 | 95.22 | 27.89 |
| Mean | **14.34** | **35.19** | **47.99** | **79.94** | **97.76** | **31.20** | **96.38** | **33.32** | **93.50** | **27.18** | **88.90** | **30.52** |
| KL | **ES** | | | | **LD** | | **TRI** | | **RA** | | **AEO** | |
|  | **Onset** | **Offset** | **Onset** | **Offset** | **Onset** | **Offset** | **Onset** | **Offset** | **Onset** | **Offset** | **Onset** | **Offset** |
| P01 | 3.27 | 93.41 | - | - | 96.78 | 21.94 | 96.92 | 22.44 | 94.84 | 15.90 | 94.34 | 18.08 |
| P02 | 8.26 | 83.70 | - | - | 96.75 | 28.63 | 96.23 | 30.31 | 91.67 | 24.57 | 91.08 | 25.08 |
| P03 | 4.96 | 92.52 | - | - | 98.45 | 26.40 | 97.77 | 26.75 | 97.57 | 23.88 | 85.63 | 34.08 |
| P04 | 2.98 | 92.47 | - | - | 99.90 | 35.46 | 93.56 | 34.67 | 94.75 | 32.69 | 92.67 | 32.25 |
| P05 | 7.68 | 89.90 | - | - | 96.97 | 22.84 | 97.72 | 25.56 | 95.14 | 19.37 | 95.49 | 21.30 |
| P06 | 6.69 | 94.45 | - | - | 98.05 | 27.14 | 96.43 | 26.55 | 94.50 | 21.80 | 86.28 | 23.28 |
| P07 | 5.18 | 88.26 | - | - | 97.36 | 26.89 | 97.25 | 27.17 | 98.31 | 29.14 | 91.14 | 31.85 |
| P08 | 4.36 | 82.71 | - | - | 98.81 | 27.41 | 97.57 | 27.54 | 98.21 | 18.90 | 95.69 | 20.21 |
| P09 | 2.37 | 93.01 | - | - | 97.47 | 20.71 | 9792 | 20.96 | 94.75 | 16.86 | 93.85 | 18.38 |
| Mean | **5.08** | **90.05** | - | - | **97.84** | **26.38** | **96.82** | **26.88** | **95.53** | **22.57** | **91.80** | **24.95** |
| NT | **ES** | | | | **LD** | | **TRI** | | **RA** | | **AEO** | |
|  | **Onset** | **Offset** | **Onset** | **Offset** | **Onset** | **Offset** | **Onset** | **Offset** | **Onset** | **Offset** | **Onset** | **Offset** |
| P01 | 3.97 | 90.39 | - | - | 97.82 | 25.51 | 98.51 | 29.72 | 95.69 | 18.63 | 97.32 | 18,68 |
| P02 | 4.45 | 84.05 | - | - | 95.59 | 31.55 | 95.29 | 33.53 | 90.54 | 27.59 | 88.63 | 29,59 |
| P03 | 4.56 | 90.39 | - | - | 98.90 | 32.89 | 97.48 | 33.84 | 98.06 | 32.99 | 98.31 | 39,18 |
| P04 | 1.04 | 88.70 | - | - | 98.11 | 34.08 | 92.47 | 34.28 | 94.99 | 31.06 | 93.85 | 38,49 |
| P05 | 4.12 | 8169 | - | - | 96.68 | 24.02 | 97.22 | 27.64 | 94.55 | 21.50 | 92.81 | 22,79 |
| P06 | 8.03 | 95.39 | - | - | 99.70 | 30.86 | 96.58 | 29.03 | 93.75 | 2422 | 91.58 | 35,41 |
| P07 | 6.29 | 94.79 | - | - | 98.24 | 33.41 | 97.67 | 34.24 | 98.21 | 34.26 | 89.69 | 32,36 |
| P08 | 4.65 | 96.33 | - | - | 97.82 | 37.51 | 96.08 | 36.08 | 97.96 | 33.88 | 96.38 | 35,69 |
| P09 | 2.89 | 94.54 | - | - | 98.11 | 22.46 | 96.14 | 22.79 | 95.04 | 18.66 | 94.24 | 19,05 |
| Mean | **4.44** | **90.70** | - | - | **97.89** | **30.25** | **96.38** | **31.24** | **95.42** | **26.98** | **93.65** | **30,14** |

**
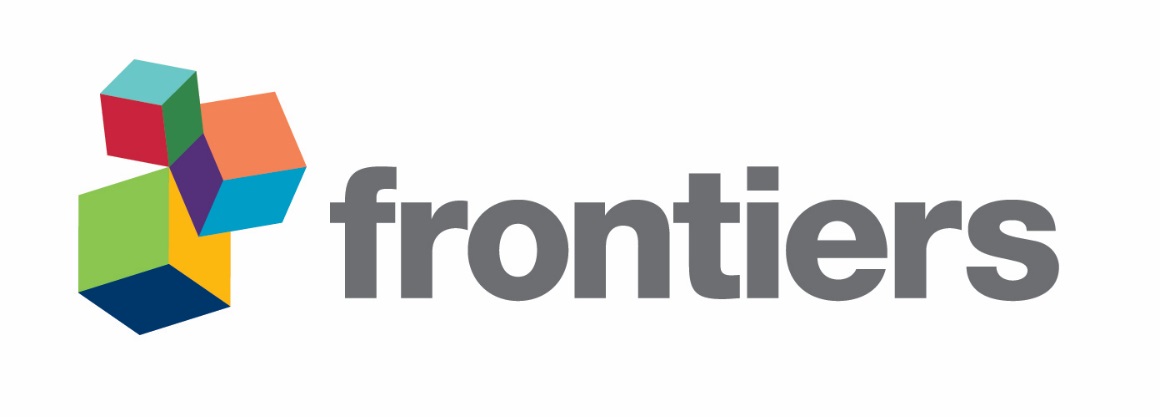
**
